# A Peptide Vaccine Design Targeting KIT Mutations in Acute Myeloid Leukemia

**DOI:** 10.3390/ph16070932

**Published:** 2023-06-27

**Authors:** Minji Kim, Kush Savsani, Sivanesan Dakshanamurthy

**Affiliations:** 1 College of Human Ecology, Cornell University, Ithaca, NY 14850, USA; 2 College of Humanities and Sciences, Virginia Commonwealth University, Richmond, VA 23284, USA; 3 Department of Oncology, Lombardi Comprehensive Cancer Center, Georgetown University Medical Center, Washington, DC 20057, USA

**Keywords:** vaccine design, acute myeloid leukemia (AML), KIT oncogene, artificial neural networks, immunoinformatics, epitopes, MHC I and MHC II molecules, epitope–MHC complexes, TCR binding, murine MHC molecules

## Abstract

Acute myeloid leukemia (AML) is a leading blood cancer subtype that can be caused by 27 gene mutations. Previous studies have explored potential vaccine and drug treatments against AML, but many were proven immunologically insignificant. Here, we targeted this issue and applied various clinical filters to improve immune response. KIT is an oncogenic gene that can cause AML when mutated and is predicted to be a promising vaccine target because of its immunogenic responses when activated. We designed a multi-epitope vaccine targeting mutations in the KIT oncogene using CD8+ and CD4+ epitopes. We selected the most viable vaccine epitopes based on thresholds for percentile rank, immunogenicity, antigenicity, half-life, toxicity, IFNγ release, allergenicity, and stability. The efficacy of data was observed through world and regional population coverage of our vaccine design. Then, we obtained epitopes for optimized population coverage from PCOptim-CD, a modified version of our original Java-based program code PCOptim. Using 24 mutations on the KIT gene, 12 CD8+ epitopes and 21 CD4+ epitopes were obtained. The CD8+ dataset had a 98.55% world population coverage, while the CD4+ dataset had a 65.14% world population coverage. There were five CD4+ epitopes that overlapped with the top CD8+ epitopes. Strong binding to murine MHC molecules was found in four CD8+ and six CD4+ epitopes, demonstrating the feasibility of our results in preclinical murine vaccine trials. We then created three-dimensional (3D) models to visualize epitope–MHC complexes and TCR interactions. The final candidate is a non-toxic and non-allergenic multi-epitope vaccine against KIT mutations that cause AML. Further research would involve murine trials of the vaccine candidates on tumor cells causing AML.

## 1. Introduction

Acute myeloid leukemia (AML) is a blood cancer subtype where an overproduction of abnormal myeloid cells causes improper development of platelets, red blood cells, white blood cells, and bone marrow failure [1]. AML is the leading acute leukemia subtype (80%) and is common among older individuals. Genetic mutations (point mutations and chromosomal translocations) are the root cause of AML. Other conditions, including myelodysplastic syndrome, aplastic anemia, myelofibrosis, Down syndrome, blood syndrome, and environmental exposures such as chemotherapy, benzene, tobacco, and radiation, have been proven to increase the risks of AML [2,3].

Common mutations that cause AML are in the genes Nucleophosmin 1 (*NPM1*), FMS-like tyrosine kinase 3 (*FLT3*), Runt-related transcription factor (*RUNX1*), and KIT, all of which are critical for hematopoiesis. The KIT gene encodes for a type III receptor tyrosine kinase that is critical for pathways involved in cell proliferation, survival, and differentiation of hematopoietic progenitor cells [4]. Synonyms for the KIT gene include c-kit, CD117, and mast/stem cell growth factor receptor (SCFR).

KIT is a proto-oncogene that can cause AML, most often core-binding factor acute myeloid leukemia (CBF-AML), and gastrointestinal stromal tumors [5,6]. CBF-AML is characterized by the chromosomal alterations t(8;21) and inv(16) [7], and 15–45% of patients suffer from mutations in the KIT gene [8]. We did not focus on CBF-AML because most of the clinically studied KIT mutations referenced in our data were not specific to CBF-AML. Still, most of the KIT mutations we analyze may be relevant to CBF-AML.

The KIT gene comprises six domains: Ig-like C2-type 1, Ig-like C2-type 2, Ig-like C2-type 3, Ig-like C2-type 4, Ig-like C2-type 5, and protein kinase. The protein kinase domain is intracellular, while the five other domains work on extracellular regions [9]. KIT mutations on the cell surface exhibit various ligand-induced activities, including ubiquitination, cell transformation, and greater sensitivity for basal tyrosine phosphorylation. Intracellular KIT mutations exhibit ligand-independent activities, including KIT activation, ubiquitination, and cell transformation [10].

AML is currently targeted with chemotherapy, drugs, and stem cell transplants [11]. Less aggressive treatments are needed because AML is an acute disease with a quick and poor prognosis, especially among the elderly. Research on CBF-AML treatments includes drug therapies such as cytarabine [12]. A successful preclinical murine trial was also conducted for a solid vaccine treatment made of polyethylene glycol and alginate [13]. Other peptide vaccine studies have targeted major mutated genes involved with AML that induce T-cell responses, including Wilms’ tumor 1 (*WT1*), proteinase 3 (*PR3*), hyaluronic acid-mediated motility receptor (*RHAMM*), and mucinone1 protein (*MUC1*). However, these vaccines have had limited success in phase II clinical trials.

The KIT gene has not been targeted with vaccines to treat AML, but trials on the gene’s effectiveness in drugs and vaccines for other conditions exist. KIT gene mutations are common targets for the treatment of various cancers because of the gene’s role in cellular functions such as hematopoiesis, carcinogenesis, and melanogenesis [14,15]. Furthermore, KIT is predicted to be a strong target for drugs and vaccines owing to its immunogenicity. C-kit ligation is associated with the release of cytokines and other pro-inflammatory mediators, and c-kit signaling can impact adaptive immunity [15]. Completed studies on treatments targeting the KIT gene include anti-drug conjugates against small cell lung cancer and a DNA vaccine targeting ligand attachment to fight tumor growth [14,16].

This study relied on inducing an immunogenic response to point mutations on the KIT oncogene that have been found in AML patients. Single amino acid changes in genes can change the chemical properties of peptide sequences and have been associated with several cancers [17]. The KIT gene was our chosen target because of its significance in cancers such as AML and its critical functions in hematopoiesis. Point mutations on KIT can lead to the development of ubiquitinated tumor-specific antigens (TSAs), which are cleaved into epitopes in proteasomes. Transporter-associated antigen processing (TAP) protein complexes direct the epitopes to bind with MHC molecules at the endoplasmic reticulum. After epitope–MHC complexes are transported to the surface of tumor cells, antigen-presenting cells help induce CD8+ and CD4+ T cells. Through their T-cell receptors (TCRs), an immune response is initiated to respond to and attack the antigen [18]. Combining CD8+ and CD4+ immune pathways may create a potent vaccine because CD4+ T cells improve the immune response of cytotoxic T cells.

Our design was a multi-epitope AML vaccine predicted to induce effective CD8+ and CD4+ T-cell responses by targeting the intracellular and extracellular domains of the KIT oncogene. We used epitopes derived from common AML-inducing point mutations in the KIT gene and overlapping CD8+ and CD4+ epitopes. Our epitope vaccine design elicits T-cell immune responses by releasing epitopes cleared through the following clinically relevant variables: HLA binding affinity, immunogenicity, antigenicity, half-life, instability, toxicity, IFNγ release, allergenicity, population coverage, and murine MHC binding affinity. CD8+ T cells respond to endogenous antigens and participate in a cytotoxic activity, while CD4+ T cells respond to exogenous antigens and are helper T cells that induce a more refined immune response. Implementing CD8+ and CD4+ epitopes into cancer vaccines is predicted to safely induce an immune response that removes tumor cells expressing the same epitopes. This study is the first to target AML with a vaccine against KIT gene mutations.

## 2. Results

**Figure 1:**
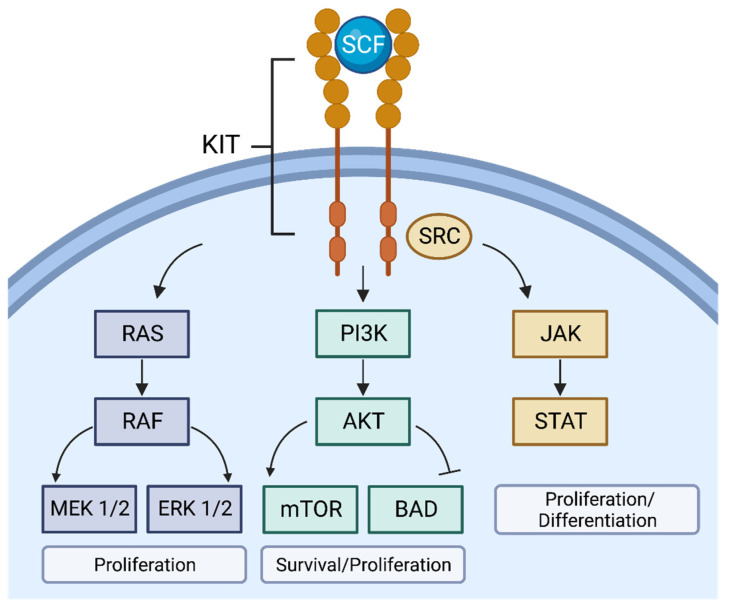
Intracellular signaling pathways that KIT is involved in. The KIT receptor tyrosine kinase can facilitate multiple signaling pathways when bound to a stem cell factor ligand. The RAS/RAF/MEK/ERK pathway guides cell proliferation. The PI3K-Akt pathway helps determine cell survival and proliferation. The JAK-STAT pathway plays a role in cell proliferation and differentiation/development (Created with BioRender.com accessed on 28 July 2022).

We targeted AML-inducing KIT mutations because of the gene’s critical roles in hematopoietic cell survival, proliferation, and differentiation (Figure 1) [19,20,21,22]. When bound to a stem cell factor, KIT facilitates multiple intracellular signaling pathways, which helps maintain normal hematopoietic cell activity. KIT mutations are also involved in immunogenic responses such as cytokine release and adaptive immunity [15]. Mutations in the KIT gene can cause improper differentiation and growth in hematopoietic cells, causing harmful conditions such as AML. The KIT gene plays critical roles in tumor cell activity, but AML vaccines targeting KIT have yet to be explored. We designed a multi-epitope vaccine combining CD8+ and CD4+ epitopes that is predicted to induce a safe immune response against mutations in the KIT gene.

**Figure 2:**
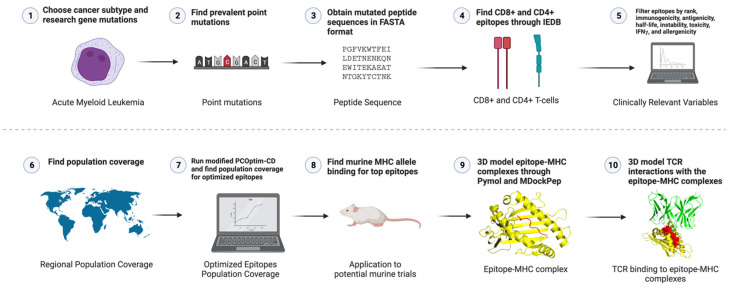
Workflow diagram of the study methodology. This process was used to develop the vaccine design; Created with BioRender.com (accessed on 18 February 2023).

Figure 2 provides a workflow of the methodology we used to design the multi-epitope AML vaccine. We started by choosing a cancer subtype, researching common gene mutations, and obtaining the mutated peptide sequences. Then we computed percentile rank, binding affinity, immunogenicity, antigenicity, half-life, instability, toxicity, IFNγ, and allergenicity values of the epitopes and filtered through the data based on specific thresholds. Optimized epitopes were obtained through a modified version of PCOptim called PCOptim-CD, which finds epitopes for optimal population coverage for both CD8+ and CD4+ datasets. Steps five through eight were repeated for both the CD8+ and CD4+ data. Finally, we modeled the top epitope–MHC complexes and their binding with TCR complexes.

### 2.1. Filtration of CD8+ Epitopes

The final CD8+ dataset included 12 ninemer epitopes filtered for rank (<10), immunogenicity (>0), antigenicity (>0.4), half-life (>1 h), toxicity (non-toxin), allergenicity (non-allergen), and instability (<40). CD8+ epitopes were not filtered for IFNγ release. IFNγ release was not prioritized for the CD8+ epitopes because CD8+ T cells are cytotoxic and less involved in releasing IFNγ than CD4+ helper T cells. Table 1 lists the top CD8+ epitopes, their respective mutations, and their binding HLA alleles. Specific values for clinically relevant variables (rank, immunogenicity, antigenicity, half-life, toxicity, allergenicity, instability, and IFNγ release) of the top CD8+ epitopes are in Supplementary Table S7.

### 2.2. Population Coverage for CD8+ Epitopes

**Figure 3:**
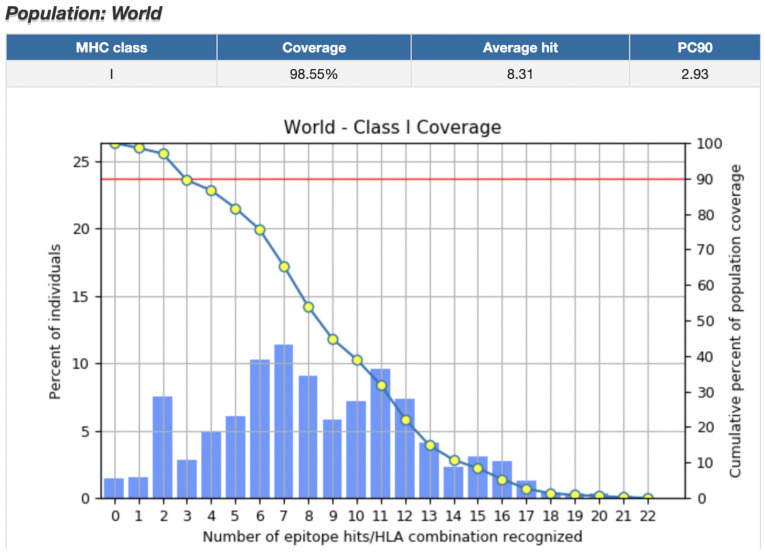
World Population Coverage for top CD8+ epitopes. World population coverage for the top CD8+ epitopes was 98.55%. Epitopes included in the calculation were filtered for percentile rank/binding affinity, immunogenicity, antigenicity, half-life, toxicity, allergenicity, and stability. Greater variety in HLA alleles resulted in higher population coverage.

Next, we determined that the world population coverage for MHC Class I binding CD8+ epitopes was 98.55% (Figure 3). Regions with high population coverage included East Asia (98.18%), Europe (99.68%), East Africa (98.18%), West Indies (98.98%), and North America (99.06%). However, Central America had an especially low population coverage of 7.76%. Population coverage for all regions is listed in Supplementary Table S1.

### 2.3. Murine MHC Binding for CD8+ Epitopes

We used the default thresholds provided by NetMHCpan-4.0 to determine strong- and weak-binding epitopes to murine MHC molecules. Strong-binding epitopes had a threshold of 0.5% and weak-binding epitopes had a threshold of 2%. There were four strong binders and six weak binders. Top CD8+ epitopes had strong binding to the murine MHC alleles H-2-Db, H-2-Dd, H-2-Kb, and H-2-Qa2. Table 1 lists strong- and weak-binding murine MHC alleles for the top CD8+ epitopes.

### 2.4. Optimized Data for CD8+ Epitopes

PCOptim-CD was used on CD8+ epitopes filtered for rank, immunogenicity, antigenicity, half-life, toxicity, allergenicity, and stability. The resulting dataset with optimal population coverage included four CD8+ epitopes (Supplementary Table S2). One optimized epitope matched a top CD8+ epitope (SNSD**I**NAAI) from Table 1. Population coverage rates of the final CD8+ epitopes and the optimized CD8+ epitopes were both 98.55% because PCOptim-CD was run on the same epitopes as the final filtered dataset.

### 2.5. Filtration of CD4+ Epitopes

The final CD4+ dataset included 21 epitopes filtered for rank (<10), immunogenicity (<50), antigenicity (>0.4), half-life (>1 h), toxicity (non-toxin), IFNγ (positive), allergenicity (non-allergen), and instability (<40). Two epitopes were 15-mers, two were 16-mers, five were 17-mers, and 12 were 18-mers. Thus, longer length epitopes had higher potency for MHC class II binding in our vaccine design. Table 2 lists the mutations, lengths, and binding HLA alleles of our top CD4+ epitopes. Specific values for clinically relevant variables (rank, immunogenicity, antigenicity, half-life, toxicity, allergenicity, instability, and IFNγ release) of the top CD8+ epitopes are in Supplementary Table S8. There were five CD4+ epitopes overlapping with top CD8+ epitopes. The C809R mutation resulted in four CD4+ epitopes (AARNILLTHGRITKI**R**DF, ARNILLTHGRITKI**R**DF, ARNILLTHGRITKI**R**DFG, ILLTHGRITKI**R**DFGLAR) overlapping with the CD8+ epitope GRITKI**R**DF from the same mutation. The K550N mutation resulted in the CD4+ epitope TYKYLQ**N**PMYEVQWK overlapping with the CD8+ epitope **N**PMYEVQWK from the same mutation.

### 2.6. Population Coverage for CD4+ Epitopes

**Figure 4:**
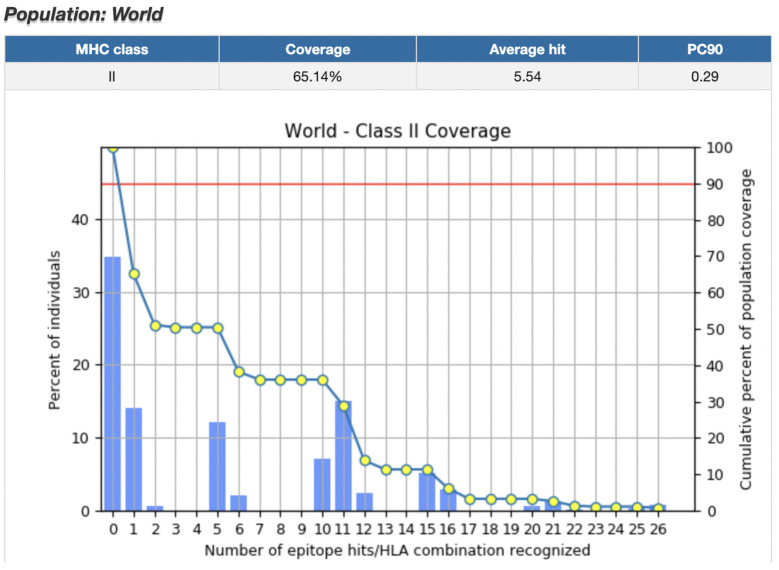
World Population Coverage for top CD4+ epitopes. World population coverage for the top CD8+ epitopes was 65.14%. Epitopes included in the calculation were filtered for percentile rank/binding affinity, immunogenicity, antigenicity, half-life, toxicity, IFNγ release, allergenicity, and stability. HLA-DRB3*02:02, HLA-DRB3*01:01, HLA-DRB1*04:05, HLA-DRB5*01:01, and HLA-DPA1*01:03/DPB1*04:01 were removed from the population coverage calculations because IEDB did not contain these alleles in their dataset.

We determined that the world population coverage for MHC Class II-binding CD4+ epitopes was 65.14% (Figure 4). Regions with highest population coverage included South Asia (62.22%), Europe (71.47%), and North America (73.34%). Regions with the lowest population coverage were Southeast Asia (29.2%), Southwest Asia (33.7%), South Africa (5.91%), and Oceania (37.6%). Population coverage for all regions is listed in Supplementary Table S3.

### 2.7. Murine MHC Binding for CD4+ Epitopes

We used the default thresholds provided by NetMHCIIpan-4.0 to determine strong- and weak-binding epitopes to murine MHC molecules. Strong-binding epitopes had a threshold of 1% and weak-binding epitopes had a threshold of 5%. There were six strong-binding epitopes and eight weak-binding epitopes. The top CD4+ epitopes had strong binding to the murine MHC alleles H-2-IEd and H-2-IEk. Table 2 lists strong- and weak-binding murine MHC alleles for the top CD4+ epitopes.

### 2.8. Optimized Data for CD4+ Epitopes

PCOptim-CD was used on CD4+ epitopes filtered for rank, immunogenicity, and antigenicity. The resulting dataset included six CD4+ epitopes (Supplementary Table S4) with a world population coverage of 99.68%. There was no overlap between the optimized epitopes and the top CD4+ epitopes from Table 2, indicating weaker results for CD4+ data compared to CD8+ data. Regions with the highest population coverage for the optimized CD4+ dataset were Northeast Asia (99.39%), South Asia (99.74%), Europe (99.98%), East Africa (99.98%), West Africa (99.94%), Central Africa (99.88%), Central America (99.5%), South America (99.99%), and Oceania (99.54%). South Africa had the lowest regional population coverage (32.1%). HLA-DRB3*01:01, HLA-DRB3*02:02, and HLA-DRB5*01:01 were disregarded from the CD4+ optimized epitopes population coverage because the IEDB dataset did not include these alleles. Supplementary Table S5 provides the world and regional population coverage of the optimized CD4+ epitopes.

### 2.9. Population Coverage for Combined Class I and Class II Molecules

We combined the final filtered dataset for Class I and Class II MHC binding epitopes and used the IEDB population coverage tool to obtain 99.49% world population coverage. Population coverage rates for specific regions are listed in Supplementary Table S6. HLA-DRB3*01:01, HLA-DRB3*02:02, HLA-DRB1*04:05, HLA-DPA1*01:03/DPB1*04:01, HLA-DRRB5*01:01, HLA-B*40:01, and HLA-A*30:01 were excluded from the combined population coverage because the IEDB dataset did not contain data for those alleles.

### 2.10. 3D Modeling for Peptide–MHC Complexes and TCR Interactions

**Figure 5:**
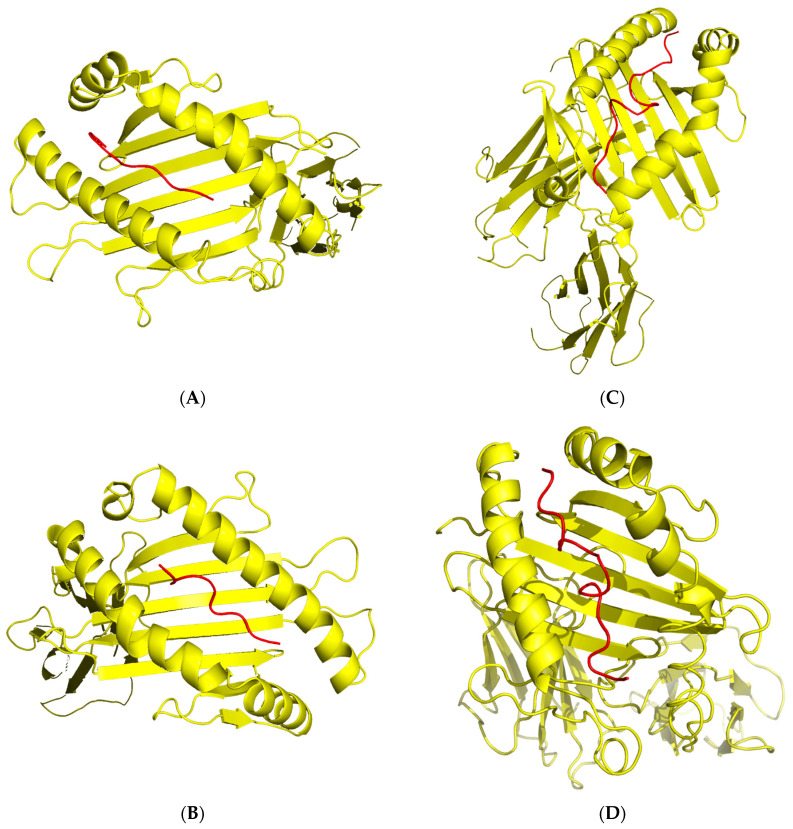
3D models for peptide–MHC complexes. SDINAAIAF binding to MHC Class I molecule HLA-A*01:01 (RCSB PDB: 6MPP) (**A**). GKSDLIVHV binding to MHC Class I molecule HLA-A*02:06 (RCSB PDB: 3OXR) (**B**). GLARYIKNDSNYVVKGN binding to MHC Class II molecule HLA-DRB1*04:01 (RCSB PDB: 5JLZ) (**C**). FGLARYIKNDSNYVVK binding to MHC Class II molecule HLA-DRB3*01:01 (RCSB PDB: 2Q6W) (**D**). Yellow represents HLA alleles, and red represents epitopes.

**Figure 6:**
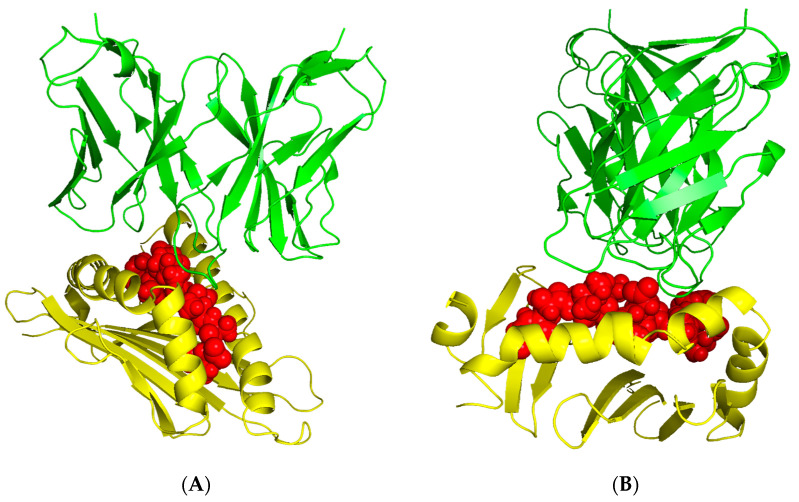
3D models for TCR binding to top epitope–MHC complexes. GKSDLIVHV epitope and MHC Class I HLA-A*02:06 binding with the A6 TCR complex (RCSB PBD: 3QH3) (**A**). GLARYIKNDSNYVVKGN epitope and MHC Class II HLA-DRB1*04:01 binding with the HA1.7 TCR complex (RCSB PDB: 4GKZ) (**B**). Green represents the TCR complex specific to the HLA allele, yellow represents the HLA allele, and red represents the epitope.

We modeled four top epitope–MHC complexes using MDockPep, CABS-dock, and PyMOL. We created 3D models for SDINAAIAF binding to HLA-A*01:01, GKSDLIVHV binding to HLA-A*02:06, GLARYIKNDSNYVVKGN binding to HLA-DRB1*04:01, and FGLARYIKNDSNYVVK binding to HLA-DRB3*01:01 (Figure 5). The 3D models for TCR interactions with peptide–MHC complexes were obtained using TCRModel (Figure 6). The A6 TCR is specific to HLA-A2 and was thus used to model an immune response to HLA-A*02:06 and the CD8+ epitope GKSDLIVHV [23]. The HA1.7 TCR is specific to HLA-DRB1*04:01 and was thus used to model an immune response to HLA-DRB1*04:01 and the CD4+ epitope GLARYIKNDSNYVVKGN [24]. Supplementary Figure S1 includes superimposed images of our epitope–MHC complexes with sample peptides from the RCSB Protein Data Bank [25] to validate the binding affinity of our epitopes to select MHC molecules.

### 2.11. 3D Modeling of Epitopes on KIT Gene

**Figure 7:**
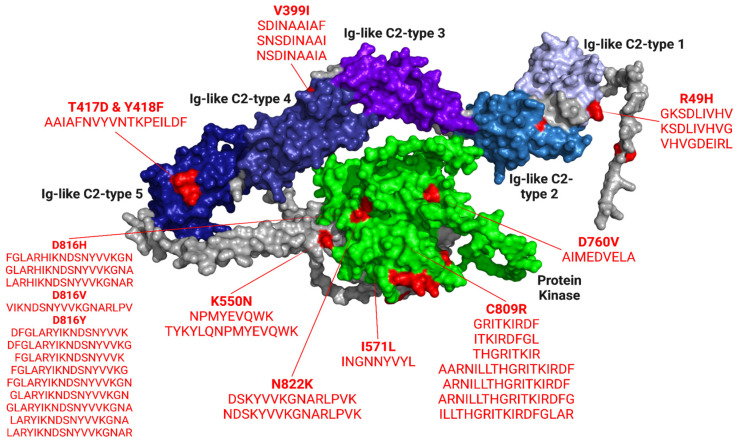
3D structure of KIT marked with the locations of our top filtered epitopes. Three of our top CD8+ epitopes (GKSDLIV**H**V, KSDLIV**H**VG, V**H**VGDEIRL) are on the Ig-like C2-type 1 domain, and three (SD**I**NAAIAF, SNSD**I**NAAI, NSD**I**NAAIA) are on the Ig-like C2-type 4 domain. Six CD8+ epitopes are on the protein kinase domain: **N**PMYEVQWK, INGNNYVY**L**, GRITKI**R**DF, ITKI**R**DFGL, THGRITKI**R**, AIMED**V**ELA. One CD4+ epitope (AAIAFNVYVNTKPEIL**DF**) is located on the Ig-like C2-type five domain. The protein kinase holds 20 of our CD4+ epitopes: FGLAR**H**IKNDSNYVVKGN, GLAR**H**IKNDSNYVVKGNA, LAR**H**IKNDSNYVVKGNAR, **V**IKNDSNYVVKGNARLPV, DFGLAR**Y**IKNDSNYVVK, DFGLAR**Y**IKNDSNYVVKG, FGLAR**Y**IKNDSNYVVK, FGLAR**Y**IKNDSNYVVKG, FGLAR**Y**IKNDSNYVVKGN, GLAR**Y**IKNDSNYVVKGN, GLAR**Y**IKNDSNYVVKGNA, LAR**Y**IKNDSNYVVKGNA, LAR**Y**IKNDSNYVVKGNAR, TYKYLQ**N**PMYEVQWK, DS**K**YVVKGNARLPVK, NDS**K**YVVKGNARLPVK, AARNILLTHGRITKI**R**DF, ARNILLTHGRITKI**R**DF, ARNILLTHGRITKI**R**DFG, ILLTHGRITKI**R**DFGLAR.

We selected a total of 33 CD8+ and CD4+ epitopes based on the filters: binding affinity/percentile rank, immunogenicity, antigenicity, half-life, toxicity, IFNγ release, allergenicity, and stability (12 CD8+ epitopes and 21 CD4+ epitopes). The KIT gene’s protein kinase domain, which affects intracellular signaling pathways, holds 26 of our top epitopes. Figure 7 locates our top epitopes in a 3D model of the KIT gene.

## 3. Discussion

There is limited research on treatments for AML that target the KIT gene. Instead, peptide vaccines and dendritic cell vaccines have targeted other tumor-associated antigens (TAAs), including WT1, PR3, RHAMM, and MUC1. Limited MHC allele interactions with epitopes have been tested in WT1 vaccines, indicating potentially low population coverage. Additionally, a WT1 vaccine restricted in HLA-A*02 had no immunological significance in its phase II clinical trial owing to minimal vaccine benefits and low sample size. A vaccine targeting OCV-501 with MHC class II molecules resulted in insignificant immunological improvements in its phase II clinical trial. TAAs are less effective than TSAs in eliciting safe immune responses to cancer cells. TSAs are only present in cancer cells and have a higher affinity to MHC molecules and TCRs, making them better candidates for anticancer vaccines. TAAs are more widely studied, but their potential toxicity and lack of specificity for tumors indicate that targeting TSAs may be an improved approach. Clinical trials with TSA-based anticancer vaccines have also been successful [18]. Further research is needed to treat AML patients with vaccines targeting TSAs, but existing trials have shown the potential use of peptide vaccines in treating AML [26].

Clinical trials for CD8+ and CD4+ epitope vaccines against AML exist, but with limited success. One such vaccine targeting the WT1 gene reached phase II of clinical trials but did not develop strong immunological memory. We addressed this issue in our vaccine design by only selecting epitopes with high antigenicity scores. However, another vaccine targeting mutated WT1 peptides resulted in improved survival. Future trials for AML vaccines must prioritize targeting TSAs instead of TAAs to ensure proper and safe immune responses [26]. In this study, we targeted the proto-oncogene KIT and identified top epitopes predicted to elicit safe immunogenicity by selecting those with high binding affinity, immunogenicity, antigenicity, half-life, toxicity, IFNγ release, allergenicity, and population coverage.

Current studies on treatments for AML that target the KIT gene emphasize drug therapy, such as combined treatment with nilotinib and chemotherapy [27] and midostaurin on patients with (8;21) translocation AML. Patients in these studies had mutations in the KIT or FLT3-ITD genes, and similar to our study, the effects of midostaurin are being observed on mut-KIT8 and mut-KIT17 [28].

Our vaccine design follows in silico methods predicted to safely induce CD8+ and CD4+ immunogenic responses. We demonstrated predicted vaccine efficacy by filtering epitopes through clinically relevant variables such as immunogenicity, antigenicity, toxicity, and allergenicity, to obtain top epitopes. Designing vaccines through bioinformatics offers a quick and cost-effective method of developing anti-cancer treatments before murine or pre-clinical trials. We identified four CD8+ and six CD4+ epitopes that were strong binders to murine MHC molecules, demonstrating potential use of our vaccine design in further research including murine trials.

We filtered out many potential CD8+ and CD4+ epitopes because of low immunogenicity scores. In the CD8+ dataset, 50% of epitopes that passed the percentile rank filter also passed the immunogenicity filter. All top CD8+ epitopes failed to pass the IFNγ filter because IFNepitope was only developed for CD4+ epitopes. Thus, the IFNγ filter was disregarded for MHC I binding molecules. For the CD4+ dataset, IFNγ and allergenicity filtered out most of the epitopes in addition to immunogenicity.

Our vaccine design was strengthened by the five CD4+ epitopes overlapping with the top CD8+ epitopes. Overlapping epitopes emphasizes their strength and our vaccine’s potential to elicit high immunogenic responses involving both cytotoxic and helper T cells. Population coverage for overlapping epitopes alone remains low, but the two potential immunogenic pathways that may be induced by the overlapping CD8+ and CD4+ epitopes indicate high potency for attacking cancerous cells. Further research on increasing population coverage of overlapping epitopes can help improve the vaccine’s effectiveness. Additionally, combined usage of CD8+ and CD4+ epitopes increase the likelihood of stability despite the short peptide lengths of CD8+ epitopes. CD8+ epitopes were limited to 9-mers, but CD4+ data included epitopes of up to 18-mers. Still, previous studies indicated that CD8+ and CD4+ immunogenic responses are inducible with vaccines using 9- or 10-mer peptides in patients with solid tumors [29].

The protein kinase domain of the KIT gene held 26 out of 33 top CD8+ and CD4+ epitopes. Each epitope was critical for our vaccine design. Still, the intracellular signaling pathways that the KIT gene is involved in, such as those outlined in Figure 2**,** are mainly instigated in the protein kinase domain. The protein kinase’s critical role in hematopoietic cell growth, proliferation and development makes the domain an important location for our top epitopes. Cancerous activities caused by mutations in the protein kinase domain can be primarily targeted by having most of our target mutations in this domain. Our study was unique in targeting a proto-oncogene for which not many have studied AML vaccine therapies. The 33 combined CD8+ and CD4+ epitopes induced a population coverage of 99.49%, ensuring that our vaccine may effectively improve AML prognosis for a large population. For both CD8+ and CD4+ epitopes, we determined population coverage based on HLA alleles that the peptides could bind to and the frequency of those alleles among various regions worldwide. High population coverage was optimal because more patients could effectively be treated with the vaccine. However, regions including Central America had lower population coverage for CD8+ and CD4+ epitopes. Large differences in population coverage such as between Central America and Europe were due to varying frequencies of HLA alleles in different populations. Each population has a unique frequency of HLA alleles, so the potency of our epitope design varies by region. In Central America, frequent HLA alleles include A*02:06:01, A*02, DQA1*05:01, and A*02:02 [30]. However, frequent alleles in Central America, such as DQA1*05:01, were still included in our top epitopes. This indicated that other discrepancies in the region’s genetic makeup may have caused lower population coverage in this region. Vaccine design methods can be improved by filtering for top epitopes that specifically bind to alleles prevalent in regions with low population coverage found in our data to maximize efficacy. Limitations in IEDB’s allele dataset also resulted in lower population coverage for certain regions, primarily with the CD4+ dataset.

Our vaccine design would be the most effective on AML patients within Asia, Europe, and North America, which included regions with the highest population coverage. HLA alleles that our top epitopes bind to were more prevalent in these regions. AML is most reported in North America, Western Europe, and South Asia, which validates our vaccine design, as our targeted population would be the most reactive to our vaccine [31].

Our data were weakly validated for population coverage owing to the minimal overlap between final epitope datasets and optimized epitopes from PCOptim-CD. However, PCOptim-CD was not as effective in our vaccine design as compared to other datasets—when PCOptim-CD was used on epitope data for a vaccine design targeting the HRAS gene for squamous cell carcinoma, the optimized dataset contained six epitopes [32]. PCOptim-CD analysis on CD8+ epitopes filtered for rank and immunogenicity only resulted in one optimal epitope. This demonstrated the high quality of our epitopes in the inputted dataset because it showed that maximum population coverage could be obtained with one epitope. However, to find more optimized epitopes, every filter had to be applied to the inputted data, making the optimized CD8+ epitope population coverage identical to that of the top CD8+ epitopes. Only one epitope from the optimized dataset matched one of our top CD8+ epitopes. Additionally, none of the epitopes in the CD4+ optimized dataset matched the top CD4+ epitopes. Therefore, population coverage of the CD4+ epitope dataset was weaker than that of CD8+. With CD4+ epitopes having a lower population coverage and less validity from PCOptim-CD, CD4+ T-cell response to our vaccine design was weak.

Peptide vaccine designs are a cost-effective method of developing treatments to target tumors and/or viruses. Computational methods also allow for large protein datasets to be quickly tested for vaccine efficacy. When compared to in vitro and in vivo studies, in silico methods are unable to reflect direct testing with living cells. To address these challenges, tools for in silico vaccine studies are constantly being developed to form optimal vaccine designs. IntegralVac is an example of this, where MHCSeqNet, DeepVacPred, and hemolytic/non-hemolytic peptide predictors were combined to improve vaccine design accuracy and safety [33]. Our data can also be used for future research to develop immunoinformatic methods to strengthen our epitope design.

### Limitations of the Study

Compared to the population coverage of the CD8+ epitopes, the CD4+ epitopes had low coverage. Additional mutations were filtered through to find more epitopes, including combination mutations with double missense, but population coverage remained low. One potential reason was that IEDB had limitations in their HLA allele dataset—a few alleles in the final CD4+ dataset were excluded from the population coverage calculation. For example, HLA-DRB3*02:02 was not included in the CD4+ population coverage, but the allele could bind to 16 of the final CD4+ epitopes. HLA-DRB3*01:01 was also excluded from the population coverage but could bind to 12 final CD4+ epitopes. Thus, the accuracy of CD4+ population coverage was limited owing to the IEDB database.

CD4+ epitope population coverage may have also been low because studies show that the KIT gene does not often interact with CD4+ T cells [34]. The KIT gene is involved in CD8+ T-cell immunodominance, but the gene was not expressed in the presence of CD4+ T cells. KIT genes can induce CD4+ T-cell immune responses, but KIT gene expression is less involved with CD4+ T cells than CD8+ T cells. Past experiments that found minimal interaction with the KIT gene and CD4+ T cells indicated why population coverage may have been low. Furthermore, because most of our mutations were on the intracellular protein kinase domain of the KIT gene, CD8+ T cells are more susceptible to being instigated, as CD8+ T cells respond to endogenous antigens, while CD4+ T cells mainly respond to exogenous antigens.

## 4. Materials and Methods

### 4.1. Finding Prevalent Point Mutations on the KIT Gene

**Figure 8:**
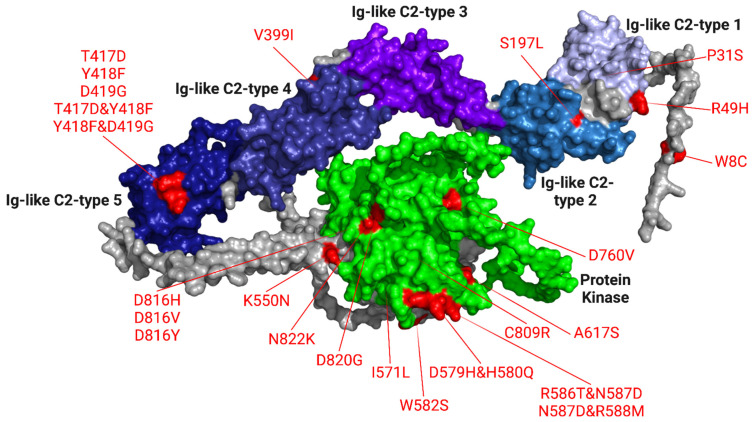
3D structure of KIT with the locations of point mutations used in our study. Two mutations are on the Ig-like C2-type 1 domain (P31S, R49H), one mutation is on the Ig-like C2-type 2 domain (S197L), and one mutation is on the Ig-like C2-type 4 domain (V399I), and five mutations are on the Ig-like C2-type 5 domain (T417D, Y418F, D419G, T417D and Y418F, Y418F and D419G). The protein kinase domain holds 14 of the mutations we used in our study (K550N, D816H/V/Y, D820G, I571L, N822K, D579H and H580Q, R586T and N587D, N587D and R588M, C809R, A617S, D760V). The AlphaFold Protein Structure Database (https://alphafold.ebi.ac.uk/entry/P10721, (accessed on 31 July 2022) [9] was used to obtain the whole KIT gene structure, and UniProt was used to identify the domains [38].

Common mutations of the KIT gene that cause AML are located on exons 17 and 8, and D816V is the most prevalent [1,35]. Mutations were chosen based on prevalence—CoDing Sequence (CDS) mutations of alanine to threonine were present in 48.20% of samples compiled in the COSMIC database. We used Y418F and D816V in this study. The CDS mutation of glycine to threonine was present in 15.51% of samples, including the point mutations W8C and D816Y used in our study. The CDS mutation of glycine to cysteine was present in 11.91% of samples, which included the point mutation D816H used in our study. Lastly, the CDS mutation of threonine to glycine was present in 9.14% of samples, including the point mutation N822K used in our study [36]. The most common mutations were located at point 816 on aspartic acid [36]. Additional point mutations found in past clinical trials [1,4,36], as well as those found in the COSMIC database (https://cancer.sanger.ac.uk/cosmic, (accessed on 2 June 2022)) [37] were used to obtain mutated KIT gene sequences. Figure 8 shows where the 24 mutations we observed are located on the KIT gene.

### 4.2. Identifying Mutated Sequences

The “mast/stem cell growth factor receptor Kit” peptide sequence was obtained in FASTA format using UniProt (https://www.uniprot.org/, (accessed on 31 July 2022) [39]. Mutated peptide sequences were determined based on the point mutations labeled in Figure 1.

### 4.3. MHC Class I Binding Epitope Prediction

9-mer CD8+ epitopes for each point mutation were obtained using the IEDB T Cell Epitope Prediction Tool with MHC I Binding (http://tools.iedb.org/mhci/, (accessed on 7 June 2022) [40]. The prediction tool was trained to predict binding affinity for top HLA alleles in humans using binding affinity and eluted ligand data. IEDB calculated a percentile rank for each epitope’s binding affinity to 27 HLA alleles: HLA-A*01:01, HLA-A*02:01, HLA-A*02:03, HLA-A*02:06, HLA-A*03:01, HLA-A*11:01, HLA-A*23:01, HLA-A*23:01, HLA-A*24:02, HLA-A*26:01, HLA-A*30:01, HLA-A*30:02, HLA-A*31:01, HLA-A*32:01, HLA-A*33:01, HLA-A*68:01, HLA-A*68:02, HLA-B*07:02, HLA-B*08:01, HLA-B*15:01, HLA-B*35:01, HLA-B*40:01, HLA-B*44:02, HLA-B*44:03, HLA-B*51:01, HLA-B*53:01, HLA-B*57:01, HLA-B*58:01. IEDB derived the percentile rank by comparing IC_50_ values of each peptide in the protein sequence with the IC_50_ values of other peptides found in the SWISSPROT database [41]. Lower percentages (above 0%) indicated higher binding affinity, and a maximum threshold of 10% was used for this filter.

Strong and stable epitope candidates were determined based on a variety of clinically relevant variables, including percentile rank (binding affinity), immunogenicity, antigenicity, half-life, instability, isoelectric point, aliphatic index, GRAVY score, toxicity, IFNγ release, and allergenicity. Only epitopes that passed these filters (IFNγ was disregarded for CD8+) were presented as top epitopes for our vaccine design.

Each epitope that passed the percentile rank filter was tested for immunogenicity using the IEDB Class I Immunogenicity tool (http://tools.iedb.org/immunogenicity/, (accessed on 13 June 2022) [42]. IEDB trained the tool to identify immunogenicity through a study of 600 immunogenic and 181 non-immunogenic peptide–MHC complexes. Further training included analysis of non-anchor positions (positions 4–6) in determining positions of high interaction with T-cell receptors (TCRs). Higher scores indicated greater immunogenicity of the epitopes, and the minimum threshold was set to 0 [43].

Antigenicity for top immunogenic epitopes was determined by VaxiJen v2.0 (http://www.ddg-pharmfac.net/vaxijen/VaxiJen/VaxiJen.html, (accessed on 13 June 2022) [44]. VaxiJen is up to 89% accurate and was developed with auto-cross covariance (ACC), turning protein sequences into vectors representing principal amino acid properties. VaxiJen v2.0 provides datasets for five organisms: bacteria, viruses, tumors, parasites, and fungi—we used tumors for our dataset. The minimum threshold used for antigenicity was 0.4—when VaxiJen was developed and tested on viral antigens, a threshold of 0.4 had 70% accuracy for external validation [44,45]. VaxiJen has been tested on multiple in silico vaccine designs, one of which identified T-cell and B-cell epitopes targeting the SARS-COV2 S protein [46].

Half-life, instability, isoelectric point, aliphatic index, and GRAVY score were determined through ProtParam (https://web.expasy.org/protparam/, (accessed on 14 June 2022). To calculate half-life, ProtParam analyzed each epitope’s N-terminal amino acid [47]. Amino acids in mammals have a minimum half-life of 0.8–1 h. Thus, we used one hour as the minimum threshold for half-life [48]. ProtParam calculates the instability index based on dipeptides. ProtParam trained the program to calculate instability using a study of 400 dipeptides in test tubes that were given weight values based on dipeptides of known stable and unstable proteins. A maximum threshold of 40 was used by ProtParam and our study to distinguish instability [49,50].

While the epitopes were not filtered for isoelectric point, aliphatic index, and GRAVY score, these values demonstrate the physicochemical properties of our top epitopes. Isoelectric point indicates the pH when a peptide reaches a neutral charge [51]. The aliphatic index was calculated by ProtParam based on the volume of aliphatic side chains (alanine, valine, leucine, and isoleucine) in the epitopes. A higher aliphatic index indicates higher thermostability. The GRAVY (grand average of hydropathy) score reveals the hydropathy of peptides, with higher scores indicating higher hydrophobicity [49].

Toxicity was obtained with ToxinPred (https://webs.iiitd.edu.in/raghava/toxinpred/, (accessed on 14 June 2022) [52]. Toxicity is determined based on SVM scores, which ToxinPred calculates based on the amino acid and dipeptide composition, binary profile pattern, and motif-based profile. The main training dataset used to develop ToxinPred included 1805 toxic peptides and 3593 non-toxic peptides. Performance on the main training dataset resulted in 93.92% maximum accuracy from the amino acid-based SVM model, 94.50% accuracy from the dipeptide-based SVM model, and 91.63% accuracy from the binary profile-based SVM model [53].

IFNγ release was tested using IFNepitope (http://crdd.osdd.net/raghava/ifnepitope/, (accessed on 14 June 2022) [54]. IFNepitope determines IFNγ release with an accuracy of 82.10% based on motifs likely to release IFNγ. IFNepitope obtained 10,433 CD4+ epitopes from IEDB to develop the dataset—3705 resulted in positive IFNγ release, and 6728 resulted in negative IFNγ release [55]. The IFNγ filter was disregarded for MHC class I molecules because IFNepitope was only developed using MHC class II molecules. However, results were still obtained for MHC class I molecules.

Allergenicity was determined using AllerTOP v2.0 (https://www.ddg-pharmfac.net/AllerTOP/, (accessed on 14 June 2022) [56]. AllerTOP v2.0 also uses ACC to develop uniform vectors from proteins. Datasets in AllerTOP v2.0 were tested against known allergenic and non-allergenic peptides. Filtering out allergenic epitopes helps design a safe vaccine because certain proteins can induce abnormal immune responses, such as rashes, sneezing, and mucous membrane swelling [57].

Population coverage was calculated using the IEDB epitope analysis tool “Population Coverage” (http://tools.iedb.org/population/, (accessed on 21 June 2022)). We used population coverage to determine our vaccine’s effectiveness on the world population and on the populations of 16 regions: East Asia, Northeast Asia, South Asia, Southeast Asia, Southwest Asia, Europe, East Africa, West Africa, Central Africa, North Africa, South Africa, West Indies, North America, Central America, South America, and Oceania. We observed population because HLA type representation varies by population and ethnicity, and maximum coverage is ideal for a vaccine design [58].

### 4.4. MHC Class II Binding Epitope Prediction

We used the same method for filtering through the CD4+ epitope dataset as we did for the CD8+ epitopes. However, percentile rank/binding affinity and immunogenicity were calculated with different tools. Percentile rank/binding affinity was obtained using the MHC II Binding Prediction tool on IEDB (http://tools.iedb.org/mhcii/, (accessed on 4 July 2022)) [59]. IEDB included 27 HLA alleles, and epitopes of length 12–18 mers were obtained. The HLA alleles studied for MHC class II molecules were HLA-DRB1*-1:-1, HLA-DRB1*03:01, HLA-DRB1*04:01, HLA-DRB1*04:05, HLA-DRB1*07:01, HLA-DRB1*08:02, HLA-DRB1*09:01, HLA-DRB1*11:01, HLA-DRB1*12:01, HLA-DRB1*13:02, HLA-DRB1*15:01, HLA-DRB3*01:01, HLA-DRB3*02:02, HLA-DRB4*01:01, HLA-DRB5*01:01, HLA-DQA1*05:01/DQB1*02:01, HLA-DQA1*05:01/DQB1*03:01, HLA-DQA1*03:01/DQB1*03:02, HLA-DQA1*04:01/DQB1*04:02, HLA-DQA1*01:01/DQB1*05:01, HLA-DQA1*01:02/DQB1*06:02, HLA-DPA1*02:01/DPB1*01:01, HLA-DPA1*01:03/DPB1*02:01, HLA-DPA1*01:02/DPB1*04:01, HLA-DPA1*03:01/DPB1*04:02, HLA-DPA1*02:01/DPB1*05:01, HLA-DPA1*02:01/DPB1*14:01. A percentile rank threshold of 10% was kept.

Immunogenicity was determined using the IEDB CD4+ T cell immunogenicity prediction tool (http://tools.iedb.org/CD4episcore/, (accessed on 4 July 2022)) [60]. This tool calculated an IEDB-recommended combined score, which is the combination of each epitope’s immunogenicity and their HLA binding prediction scores. Combined scores had a maximum area under the ROC curve (AUC) score of 0.71 with a training dataset of 530 immunogenic peptides and 1758 non-immunogenic peptides [61]. We calculated the percent of MHC class I binding epitopes that passed the immunogenicity threshold of 0 to determine a threshold for CD4+ epitope immunogenicity. Half of the CD8+ epitopes passed the immunogenicity filter, so a maximum combined score of 50 (out of 100) was used for the CD4+ epitope immunogenicity threshold. A lower combined score indicated a better T-cell response.

### 4.5. Obtaining Optimized Population Coverage with PCOptim-CD

The final epitopes had several overlapping amino acid sequences. We developed PCOptim-CD to find an optimized epitope dataset with maximal population coverage to reduce redundancy in epitope selection. The original program, PCOptim, was only designed for CD8+ datasets. The modified version, PCOptim-CD, was programmed to obtain the optimized epitopes for the CD4+ dataset as well. PCOptim-CD (Supplementary Figure S1) was based on the console version, called PopCoverageOptimization. Therefore, it is text-based rather than GUI-based, and instructions for using the program can be found in the comments of the Java code.

Epitopes and their MHC-restricted alleles for optimal population coverage were obtained using PCOptim-CD [32]. We used CD8+ epitopes filtered by rank, immunogenicity, antigenicity, half-life, instability, toxicity, and allergenicity to find multiple optimal CD8+ epitopes. We used CD4+ epitopes filtered by rank, immunogenicity, and antigenicity to obtain the optimized CD4+ dataset. PCOptim-CD allowed us to identify epitopes from our full dataset that were likely to have optimal population coverage.

### 4.6. Murine MHC Binding

Strong- and weak-binding CD8+ epitopes to murine MHC molecules were identified using NetMHCpan-4.0 for peptide-MHC class I binding (https://services.healthtech.dtu.dk/service.php?NetMHCpan-4.0, (accessed on 28 June 2022) [62]. NetMHCpan-4.0 used artificial neural networks (ANNs) to give results for the following murine MHC alleles: H-2-Db, H-2-Dd, H-2-Kb, H-2-Kd, H-2-Kk, H-2-Ld, H-2-Qa1, and H-2-Qa2. For CD4+ epitopes, NetMHCIIpan-4.0 for peptide–MHC class II binding was used (https://services.healthtech.dtu.dk/service.php?NetMHCIIpan-4.0, (accessed on 19 July 2022) [63], which gave results for the following murine MHC alleles: H-2-IAu, H-2-Ied, and H-2-IEk.

### 4.7. Three-Dimensional (3D) Modeling of Peptide–MHC Complex and TCR Interactions

We found PDB files for four HLA alleles that were restricted by several of our top epitopes on the RCSB Protein Data Bank (https://www.rcsb.org/, (accessed on 21 July 2022) [25]. HLA alleles were chosen based on the MHC restrictions presented for each CD8+ and CD4+ epitope listed by IEDB. Using MDockPeP (https://zougrouptoolkit.missouri.edu/mdockpep/, (accessed on 21 July 2022) [64,65,66] and CABS-dock [67], select epitopes from our final dataset were attached to binding grooves of HLA alleles to create four 3D models of peptide–MHC complexes. Both MDockPeP and CABS-dock generate top-scoring docking models with minimal binding energy. TCRModel (https://tcrmodel.ibbr.umd.edu/rtcrex/TCRSDM6_180718_160348, (accessed on 31 July 2022) [68] was used to create 3D models of TCR complex interactions with our peptide–MHC complexes. All 3D models were edited with PyMOL.

## 5. Conclusions

Several studies have investigated how to treat AML, including drug therapies, combination therapy (drugs and chemotherapy), stem cell transplants, and vaccines. However, many treatments, including AML vaccines that target the KIT gene, remain unexplored. The purpose of this study was to develop a vaccine design for AML using in silico methods that target missense mutations on the KIT oncogene. We applied several clinically relevant variables to our vaccine epitopes, including percentile rank, immunogenicity, antigenicity, half-life, toxicity, IFNγ release, allergenicity, and stability, to ensure the vaccine’s safety and effectiveness. Then, population coverage demonstrated the broadness of our vaccine design’s potential. Using this method, we found 12 CD8+ and 21 CD4+ epitopes from mutated KIT peptide sequences that can be implemented in a vaccine and potentially used in murine trials. The 12 CD8+ epitopes were immunogenic, antigenic, non-toxic, non-allergenic, and had long half-lives. In comparison, the 21 CD4+ epitopes were immunogenic, antigenic, non-toxic, non-allergenic, have long half-lives, and release IFNγ. The CD8+ epitopes had a high population coverage of 98.55%, while the CD4+ epitopes had a lower population coverage of 65.14% owing to limitations in our tools’ datasets and minimal interactions between the KIT gene and CD4+ T-cells. PCOptim was modified into PCOptim-CD to analyze both CD8+ and CD4+ datasets for optimized population coverage. There was minimal overlap between the final filtered epitopes and the optimized epitopes from PCOptim-CD, proving that further research is needed to develop a stronger dataset with greater validity. The four CD8+ and six CD4+ epitopes that were strong binders to murine MHC alleles indicated that our results can lead to preclinical studies with vaccine trials on murine models. We designed a vaccine predicated to be safe and effective through in silico methods to help improve treatments for AML and develop cost-effective methods for vaccine designs before pre-clinical trials. Our data may be used to facilitate future studies in investigating the use of our vaccine design in murine and clinical trials and improving immunoinformatic tools. Murine trials with the peptide vaccine design would be the next step for advancing research on this treatment for AML. Using the top epitopes with strong binding to murine MHC molecules, hematopoietic and stem and progenitor cells from mice would be modified with genome editing in vitro. The treatment group would receive these cells intravenously (IV) in addition to radiation treatment [69] and IV-administered peptide vaccine, and the control group would receive normal saline administration. The study would include dosage testing to measure the appropriate dosage needed for the peptide vaccine. qPCR analysis may be conducted to measure the presence of the KIT gene as well as mutant KIT genes. RNA-sequence analysis would be used to measure prevalence of the single amino acid mutations found in our peptide vaccine. MHC-epitope binding complexes would be isolated with immunoprecipitation assays to confirm the success of epitope binding to target MHC allele. SCF binds to the KIT gene to induce various cellular pathways, and SCF-ELISA assay may be used to analyze antibody binding levels on KIT to assess KIT function. The results of these experiments with murine trials would determine whether the peptide vaccine can be tested further clinically.

## Figures and Tables

**Table 1:** Top CD8+ Epitopes and Murine Binding.

Mutation	Epitope	HLA Alleles	Strong H2 Allele Restriction	Weak H2 Allele Restriction
I571L	INGNNYVY**L**	HLA-A*24:02, HLA-B*08:01, HLA-A*23:01, HLA-A*68:02	H-2-Db, H-2-Dd, H-2-Kb	H-2-Ld
K550N	**N**PMYEVQWK	HLA-A*68:01, HLA-B*35:01, HLA-A*33:01, HLA-B*53:01, HLA-A*11:01, HLA-A*03:01, HLA-B*07:02	Not available	Not available
R49H	GKSDLIV**H**V	HLA-A*02:06, HLA-A*02:03, HLA-A*68:02, HLA-A*02:01, HLA-B*40:01 HLA-A*30:01, HLA-B*44:03, HLA-B*51:01, HLA-B*44:02, HLA-A*30:02, HLA-A*26:01, HLA-B*15:01	Not available	Not available
R49H	KSDLIV**H**VG	HLA-B*58:01, HLA-B*57:01, HLA-A*01:01	Not available	Not available
R49H	V**H**VGDEIRL	HLA-A*23:01, HLA-B*40:01, HLA-A*24:02, HLA-B*44:03, HLA-B*35:01, HLA-B*44:02, HLA-B*53:01	Not available	H-2-Kd
V399I	SD**I**NAAIAF	HLA-B*44:03, HLA-B*44:02, HLA-B*40:01, HLA-B*15:01, HLA-B*35:01, HLA-A*26:01, HLA-A*30:02, HLA-B*53:01, HLA-A*01:01, HLA-B*07:02, HLA-A*32:01, HLA-A*23:01, HLA-A*24:02, HLA-B*58:01	H-2-Qa2	H-2-Kk, H-2-Ld
V399I	SNSD**I**NAAI	HLA-A*68:02, HLA-B*51:01, HLA-A*02:06,HLA-B*40:01, HLA-A*30:02, HLA-A*02:03, HLA-A*26:01, HLA-B*07:02, HLA-B*58:01, HLA-A*32:01, HLA-B*44:02, HLA-B*44:03, HLA-A*01:01, HLA-B*53:01, HLA-B*35:01, HLA-A*23:01, HLA-A*24:02	Not available	H-2-Kk
V399I	NSD**I**NAAIA	HLA-A*01:01, HLA-B*51:01, HLA-A*68:02, HLA-B*35:01	Not available	H-2-Db
D760V	AIMED**V**ELA	HLA-A*02:06, HLA-A*02:01, HLA-A*02:03, HLA-A*68:02, HLA-A*30:02, HLA-A*26:01, HLA-A*01:01, HLA-A*32:01, HLA-A*11:01	Not available	Not available
C809R	GRITKI**R**DF	HLA-B*08:01, HLA-A*30:02, HLA-B*15:01, HLA-A*23:01, HLA-A*26:01, HLA-B*44:03, HLA-A*32:01, HLA-A*24:02, HLA-B*44:02, HLA-B*40:01	Not available	Not available
C809R	ITKI**R**DFGL	HLA-B*08:01, HLA-B*57:01, HLA-A*30:01, HLA-B*58:01, HLA-A*68:02, HLA-A*32:01, HLA-A*02:06, HLA-B*07:02, HLA-A*30:02, HLA-B*51:01, HLA-A*02:03, HLA-A*31:01, HLA-B*15:01, HLA-A*33:01, HLA-A*24:02, HLA-A*23:01	Not available	Not available
C809R	THGRITKI**R**	HLA-A*33:01, HLA-A*31:01, HLA-A*68:01	Not available	Not available

**Table 2:** Top CD4+ Epitopes.

Mutation	Length	Epitope	HLA Alleles	Strong H2 Allele Restriction	Weak H2 Allele Restriction
D816H	18	FGLAR**H**IKNDSNYVVKGN	HLA-DRB1*13:02, HLA-DRB3*02:02, HLA-DRB3*01:01	Not available	Not available
D816H	18	GLAR**H**IKNDSNYVVKGNA	HLA-DRB1*13:02, HLA-DRB3*02:02, HLA-DRB3*01:01	Not available	Not available
D816H	18	LAR**H**IKNDSNYVVKGNAR	HLA-DRB1*13:02, HLA-DRB3*02:02, HLA-DRB3*01:01	Not available	Not available
D816V	18	**V**IKNDSNYVVKGNARLPV	HLA-DRB1*13:02, HLA-DRB3*02:02, HLA-DRB1*08:02, HLA-DRB1*15:01	Not available	H-2-IEd
D816Y	17	DFGLAR**Y**IKNDSNYVVK	HLA-DRB3*02:02, HLA-DRB1*13:02, HLA-DRB3*01:01, HLA-DRB1*08:02, HLA-DRB1*04:01, HLA-DRB1*15:01	Not available	H-2-IEd
D816Y	18	DFGLAR**Y**IKNDSNYVVKG	HLA-DRB3*02:02, HLA-DRB1*13:02, HLA-DRB3*01:01, HLA-DRB1*08:02, HLA-DRB1*04:01, HLA-DRB1*15:01	Not available	H-2-IEd
D816Y	16	FGLAR**Y**IKNDSNYVVK	HLA-DRB3*02:02, HLA-DRB1*13:02, HLA-DRB3*01:01, HLA-DRB1*15:01, HLA-DRB1*04:01, HLA-DRB1*08:02	H-2-IEd	Not available
D816Y	17	FGLAR**Y**IKNDSNYVVKG	HLA-DRB3*02:02, HLA-DRB1*13:02, HLA-DRB3*01:01, HLA-DRB1*08:02, HLA-DRB1*04:01, HLA-DRB1*15:01	H-2-IEd	Not available
D816Y	18	FGLAR**Y**IKNDSNYVVKGN	HLA-DRB3*02:02, HLA-DRB1*13:02, HLA-DRB3*01:01, HLA-DRB1*08:02, HLA-DRB1*04:01, HLA-DRB1*15:01	Not available	H-2-IEd
D816Y	17	GLAR**Y**IKNDSNYVVKGN	HLA-DRB3*02:02, HLA-DRB1*13:02, HLA-DRB3*01:01, HLA-DRB1*08:02, HLA-DRB1*04:01, HLA-DRB1*15:01	H-2-IEd	Not available
D816Y	18	GLAR**Y**IKNDSNYVVKGNA	HLA-DRB3*02:02, HLA-DRB1*13:02, HLA-DRB3*01:01, HLA-DRB1*08:02, HLA-DRB1*04:01, HLA-DRB1*15:01	Not available	H-2-IEd
D816Y	17	LAR**Y**IKNDSNYVVKGNA	HLA-DRB3*02:02, HLA-DRB1*13:02, HLA-DRB3*01:01, HLA-DRB1*08:02, HLA-DRB1*04:01, HLA-DRB1*15:01	Not available	H-2-IEd
D816Y	18	LAR**Y**IKNDSNYVVKGNAR	HLA-DRB3*02:02, HLA-DRB1*13:02, HLA-DRB3*01:01, HLA-DRB1*08:02, HLA-DRB1*04:01, HLA-DRB1*15:01	Not available	H-2-IEd
N822K	15	DS**K**YVVKGNARLPVK	HLA-DRB3*02:02, HLA-DRB1*13:02, HLA-DRB5*01:01, HLA-DRB1*01:01, HLA-DRB1*08:02, HLA-DRB1*11:01, HLA-DRB1*15:01	H-2-IEd, H-2-IEk	Not available
N822K	16	NDS**K**YVVKGNARLPVK	HLA-DRB3*02:02, HLA-DRB1*13:02, HLA-DRB5*01:01	H-2-IEd	H-2-IEk
K550N	15	TYKYLQ**N**PMYEVQWK	HLA-DRB3*02:02 HLA-DRB1*04:05, HLA-DRB1*04:01, HLA-DPA1*01:03/DPB1*04:01	Not available	Not available
C809R	18	AARNILLTHGRITKI**R**DF	HLA-DRB1*07:01	Not available	Not available
C809R	17	ARNILLTHGRITKI**R**DF	HLA-DRB1*07:01	Not available	Not available
C809R	18	ARNILLTHGRITKI**R**DFG	HLA-DRB1*07:01	Not available	Not available
C809R	18	ILLTHGRITKI**R**DFGLAR	HLA-DRB1*07:01	Not available	Not available
T417D & Y418F	18	AAIAFNVYVNTKPEIL**DF**	HLA-DRB1*07:01, HLA-DRB3*02:02	Not available	Not available

## Data Availability

Data is contained within the article and Supplementary Material.

## Supplementary Materials

The following supporting information can be downloaded at: https://www.mdpi.com/article/10.3390/ph16070932/s1, Figure S1: Superimposed models of epitope–MHC complexes with sample peptides. SDINAAIAF binding to MHC Class I molecule HLA-A*01:01 superimposed with PDB ID: 6MPP (**A**). GKSDLIVHV binding to MHC Class I molecule HLA-A*02:06 superimposed with PDB ID: 3OXR (**B**). GLARYIKNDSNYVVKGN binding to MHC Class II molecule HLA-DRB1*04:01 superimposed with PDB ID: 5JLZ (**C**). FGLARYIKNDSNYVVK binding to MHC Class II molecule HLA-DRB3*01:01 superimposed with PDB ID: 2Q6W (**D**); Table S1: Population Coverage for CD8 Epitopes; Table S2: Optimized CD8 Epitopes; Table S3: Population Coverage for CD4 Epitopes; Table S4: Optimized CD4 Epitopes; Table S5: Population Coverage for Optimized CD4 Epitopes; Table S6: Combined Class I and Class II Population Coverage; Table S7: Top CD8+ Epitopes Clinically Relevant Variables; Table S8: Tope CD4+ Epitopes Clinically Relevant Variables.
