# Supplementary material for: A Peptide Vaccine Design Targeting KIT Mutations in Acute Myeloid Leukemia
Source: Pharmaceuticals (Basel). 2023 Jun 27;16(7):932. doi: 10.3390/ph16070932 (PMC10383192; doi:10.3390/ph16070932)
Supplement: Supplementary file 1 [file pharmaceuticals-16-00932-s001.zip › Supplementary Materials.pdf]

pharmaceuticals

MDPI

1. Supplementary Files/Tables

Supplementary Table S1. Population Coverage for CD8 Epitopes

**Region**	**Percent Population Coverage** (%)
World	98.55
East Asia	98.18
Northeast Asia	94.7
South Asia	94.73
Southeast Asia	94.56
Southwest Asia	92.5
Europe	99.68
East Africa	98.18
West Africa	95.49
Central Africa	86.04
North Africa	96.03
South Africa	93.03
West Indies	98.98
North America	99.06
Central America	7.76
South America	88.3
Oceania	94.71

Supplementary Table S2. Optimized CD8 Epitopes

**Epitope**	**HLA Alleles**
NPMYEVQWK	HLA-A*68:01, HLA-B*35:01, HLA-A*33:01, HLA-B*53:01, HLA-A*11:01, HLA-A*03:01, HLA-B*07:02
SNSDINAAI	HLA-A*68:02, HLA-B*51:01, HLA-A*02:06, HLA-B*40:01, HLA-A*30:02, HLA-A*02:03, HLA-A*26:01, HLA-B*07:02, HLA-B*58:01, HLA-A*32:01, HLA-B*44:02, HLA-B*44:03, HLA-A*01:01, HLA-B*53:01, HLA-B*35:01, HLA-A*23:01, HLA-A*24:02

ITKIRDFGL	HLA-B*08:01, HLA-B*57:01, HLA-A*30:01, HLA-B*58:01, HLA-A*68:02, HLA-A*32:01, HLA-A*02:06, HLA-B*07:02, HLA-A*30:02, HLA-B*51:01, HLA-A*02:03, HLA-A*31:01, HLA-B*15:01, HLA-A*33:01, HLA-A*24:02, HLA-A*23:01
GKSDLIVHV	HLA-A*02:06, HLA-A*02:03, HLA-A*68:02, HLA-A*02:01, HLA-B*40:01, HLA-A*30:01, HLA-B*44:03, HLA-B*51:01, HLA-B*44:02, HLA-A*30:02, HLA-A*26:01, HLA-B*15:01

Supplementary Table S3. Population Coverage for CD4 Epitopes

**Region**	**Percent Population Coverage** (%)
World	65.14
East Asia	51.04
Northeast Asia	35.03
South Asia	62.22
Southeast Asia	29.2
Southwest Asia	33.7
Europe	71.47
East Africa	47.87
West Africa	43.77
Central Africa	46.34
North Africa	56.01
South Africa	5.91
West Indies	56.3
North America	73.34
Central America	44.05
South America	44.84
Oceania	37.6

Supplementary Table S4. Optimized CD4 Epitopes

**Epitopes**	**HLA Alleles**
YGLIKSDASMTVAVKM	HLA-DRB1*04:01, HLA-DRB3*02:02, HLA-DRB1*13:02, HLA-

	DRB3*01:01, HLA-DRB1*03:01, HLA-DRB1*09:01, HLA-DPA1*02:01, HLA-DRB1*07:01, HLA-DQA1*01:02
NGNNYVYLDPTQLPY	HLA-DRB1*04:05, HLA-DRB1*01:01, HLA-DRB1*03:01, HLA-DRB3*01:01, HLA-DRB5*01:01, HLA-DRB1*04:01, HLA-DQA1*05:01, HLA-DQA1*01:01, HLA-DRB1*13:02, HLA-DRB3*02:02
PEILTFDRLVNGMLQ	HLA-DRB1*09:01, HLA-DRB1*04:05, HLA-DRB1*01:01, HLA-DRB1*11:01, HLA-DPA1*03:01, HLA-DRB1*03:01, HLA-DRB1*15:01
YTFLVSNSDINAAIAFNV	HLA-DQA1*03:01, HLA-DRB3*02:02, HLA-DQA1*01:02, HLA-DRB1*04:05, HLA-DQA1*04:01, HLA-DRB1*09:01, HLA-DRB1*04:01, HLA-DQA1*05:01, HLA-DRB1*13:02
LTYKYLQNPMYEVQWK	HLA-DRB1*04:01, HLA-DRB3*02:02, HLA-DRB1*04:05, HLA-DRB1*01:01, HLA-DPA1*01:03, HLA-DRB1*11:01
FGLARVIKNDSNYVV	HLA-DRB1*13:02, HLA-DRB3*02:02, HLA-DRB1*08:02, HLA-DRB1*15:01, HLA-DRB3*01:01, HLA-DRB1*03:01, HLA-DRB1*04:01, HLA-DRB1*11:01, HLA-DRB1*12:01

Supplementary Table S5. Population Coverage for Optimized CD4 Epitopes

**Region**	**Percent Population Coverage** (%)
World	99.68
East Asia	96.83
Northeast Asia	99.39
South Asia	99.74
Southeast Asia	94.53
Southwest Asia	89.93
Europe	99.98
East Africa	99.98
West Africa	99.94
Central Africa	99.88
North Africa	95.88
South Africa	32.1
West Indies	97.25
North America	100.0

Central America	99.5
South America	99.99
Oceania	99.54

Supplementary Table S6. Combined Class I and Class II Population Coverage

**Region**	**Percent Population Coverage** (%)
**World**	**99.49**
East Asia	99.11
Northeast Asia	96.55
South Asia	98.01
Southeast Asia	96.15
Southwest Asia	95.03
Europe	99.91
East Africa	95.19
West Africa	97.49
Central Africa	92.51
North Africa	98.25
South Africa	98.01
West Indies	99.55
North America	99.75
Central America	48.39
South America	93.54
Oceania	96.7

Supplementary Table 7. Attached in excel file.

Supplementary Table 8. Attached in excel file.

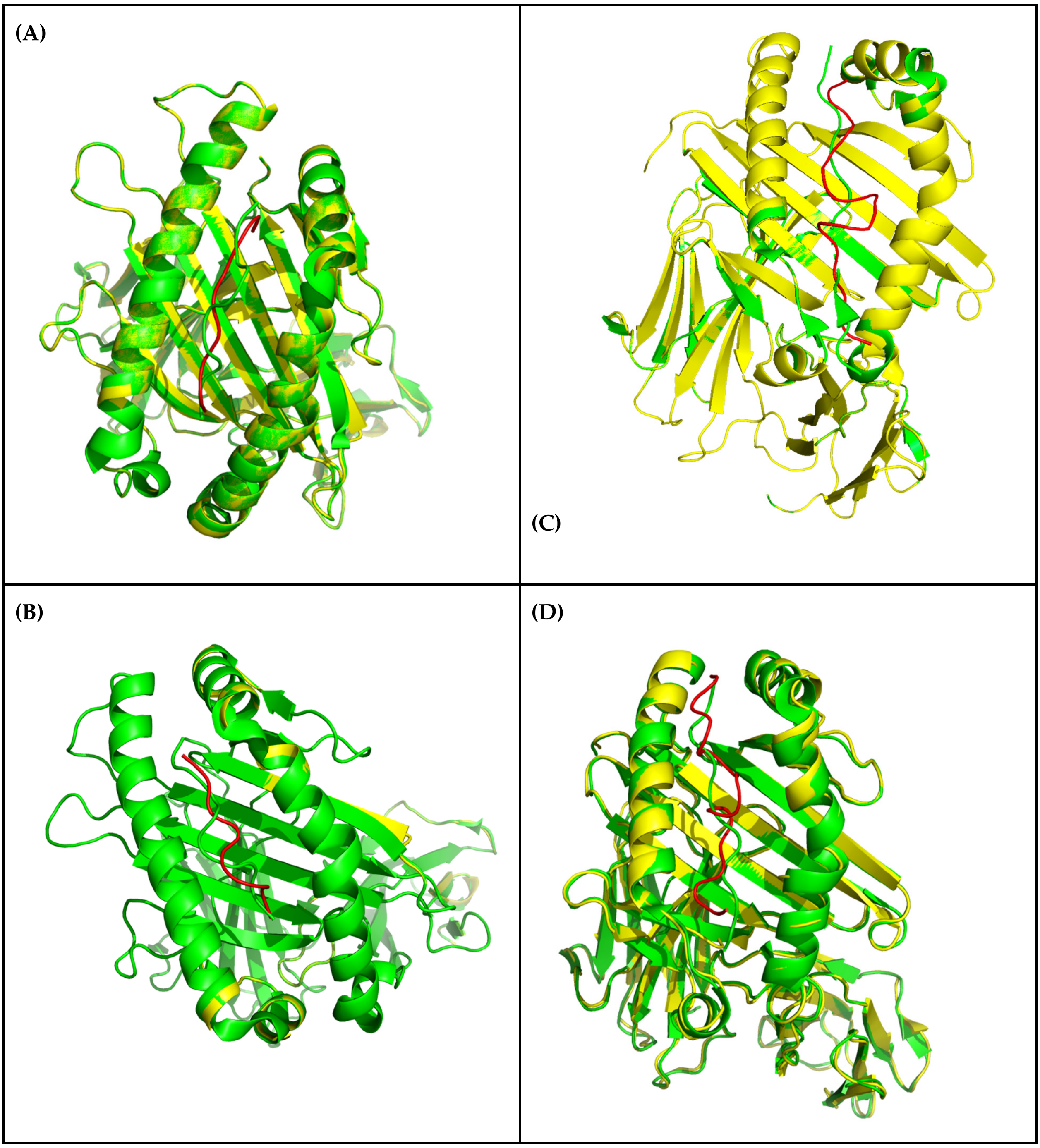

Supplementary Figure S1. ***Superimposed models of epitope-MHC complexes with sample peptides.*** *SDINAAIAF binding to MHC Class I molecule HLA-A*01:01 superimposed with PDB ID: 6MPP (**A**). GKSDLIVHV binding to MHC Class I molecule HLA-A*02:06 superimposed with PDB ID: 3OXR (**B**). GLARYIKNDSNYVVKGN binding to MHC Class II molecule HLA-DRB1*04:01 superimposed with PDB ID: 5JLZ (**C**). FGLARYIKNDSNYVVK binding to MHC Class II molecule HLA-DRB3*01:01 superimposed with PDB ID: 2Q6W (**D**).*
